# A Rat Drinking in the Dark Model for Studying Ethanol and Sucrose Consumption

**DOI:** 10.3389/fnbeh.2017.00029

**Published:** 2017-02-22

**Authors:** Joan Y. Holgate, Masroor Shariff, Erica W. H. Mu, Selena Bartlett

**Affiliations:** Institute of Health and Medical Innovation, Translational Research Institute, Queensland University of TechnologyWoolloongabba, QLD, Australia

**Keywords:** intermittent access 2-bottle choice, drinking in the dark, ethanol, sucrose, varenicline, rat, consumption model

## Abstract

**Background**: The intermittent access 2-bottle choice (IA2BC) and drinking in the dark (DID) models were developed for studying rodent binge-like consumption. Traditionally, IA2BC was used with rats and DID with mice. Recently, IA2BC was adapted to study mouse ethanol consumption. However, it is unknown whether DID is suitable for rats or if one rat model is more advantageous than another for studying binge-like consumption.

**Methods**: Male Wistar rats consumed 20% ethanol or 5% sucrose using IA2BC or DID for 12 weeks. IA2BC drinking sessions occurred on alternate days (Mondays–Fridays) and lasted 24 h, whereas DID sessions ran 4 h/day, 5 days/week (Monday–Friday). Average consumption/session, week and hour was measured. To explore DID model suitability for screening novel compounds for controlling ethanol and sucrose intake, varenicline (2 mg/kg) or vehicle was administered to DID rats.

**Results**: IA2BC rats consume more ethanol/session and similar amounts of ethanol/week than DID rats. While, IA2BC rats consume more sucrose/session and week than DID rats. Although IA2BC rats had more ethanol and sucrose access time, DID rats had greater ethanol and sucrose intake/hour. Varenicline significantly reduced ethanol and sucrose consumption in DID rats, consistent with previously published IA2BC studies.

**Conclusions**: Despite the shorter access time, the rat DID model induced higher initial intake and greater consumption/hour for both ethanol and sucrose. The shorter duration of DID sessions did not prevent detection of varenicline-induced reductions in ethanol or sucrose consumption, suggesting the DID model may be suitable for studying binge-like ethanol and sucrose consumption.

## Introduction

Harmful consumption of substances, like alcohol and sugar, remain a world-wide health problem. In 2012, 3.3 million deaths were attributed to alcohol consumption and in 2010, 3.4 million deaths to being overweight or obese, with excessive sugar consumption identified as a leading cause of body weight gain (WHO, 2014). With few effective interventions and treatments available, there remains a critical need for the development of new medications and management strategies for reducing alcohol and sugar intake. Rodent consumption models are valuable tools for examining the mechanisms underlying harmful consumption behaviors and developing new therapies. Both rodent species offer advantages and disadvantages for modeling consumption and sometimes the use of a rat model is preferable to a mouse model. For example, rats are generally larger than mice and consequentially have larger brains, allowing the study of smaller brain regions and more accurate placement of cannula, viruses and other probes. Rats also have a slower metabolic rate which expands the timeframe available to measure the effects of potential interventions (like exercise and pharmacotherapeutics) on consumption and reward seeking behaviors. Studies examining the alcohol deprivation effect (ADE) in rats generally produce more consistent results as the ADE last for up to 4 days following the reintroduction of ethanol compared to 1 day in mice (Vengeliene et al., 2014). Additionally, mouse ethanol consumption behaviors differ from rat behaviors. Specifically, mice consuming ethanol using the drinking in the dark model (DID) model, compared to rats using the intermittent access 2-bottle choice (IA2BC) model, display greater insensitive to quinine adulteration of ethanol (a test designed to measure ethanol dependence) making it difficult to dissect the natural compulsive behaviors of the mouse from compulsive behaviors associated with ethanol exposure (Hopf and Lesscher, 2014). However, transgenic mice are more readily available than transgenic rats for isolating genes and signaling pathways involved in modulating consumption behaviors. Hence, it is essential for the researcher to have a diverse and adaptable range of tools and models available for studying all aspects of consumption and related behaviors in both species.

The recent development of two new preclinical rodent models of consumption (DID and IA2BC) has improved our ability to screen and identify novel pharmacotherapeutic targets for the management of consumption behaviors. These models were originally designed to mimic clinically relevant aspects of human addiction defined in the Diagnostic and Statistical Manual (American Psychiatric Association, 2013). Generally, these models are considered to model binge-like consumption as ethanol consumption was demonstrated to be greatest in the period immediately following the start of the drinking sessions (Rhodes et al., 2005). Typically, to more closely model humans, the rodent commences ethanol consumption using these models during adolescence. Many alcoholics report their first ethanol experiences in their teens, a period where the perception of negative properties of alcohol is reduced, leading to a greater propensity for binge consumption and increased risk for developing alcohol use disorders (AUDs) later in life (Clapper and Lipsitt, 1992; Bonomo et al., 2004; Wells et al., 2004). Additionally, preclinical research indicates that exposure to ethanol commencing in adolescence keeps the brain in an adolescent-like state in adulthood (Nielsen et al., 2012b), providing insight into why commencing ethanol consumption in adolescents results in greater ethanol consumption compared to those commencing ethanol consumption as adults (Vetter et al., 2007). Sucrose, on the other hand, was traditionally used in these models as a natural reward control, to demonstrate the effects of the novel compound were specific to ethanol consumption and not consumption in general. However, with the recent accumulation of evidence supporting the addictive properties of sugar and its ability to modulate the same reward pathway that drugs of abuse act upon (for reviews, see Avena et al., 2008; Benton, 2010), the IA2BC and DID models are increasingly being used to study sucrose consumption (for examples, see Galic and Persinger, 2002; Steensland et al., 2010; Mangabeira et al., 2015; Shariff et al., 2016).

Two-bottle choice (2BC) models, like the IA2BC and DID models are often chosen for studying consumption behaviors as they are technically simple and accessible to most researchers, and generally facilitate levels of consumption that are considered clinically relevant to the targeted human condition (for reviews, see McBride and Li, 1998; Carnicella et al., 2014; Griffin, 2014). They also provide the rodent the opportunity to choose how much ethanol or sucrose they wish to consume—a desirable model quality since the loss of control over the ability to choose not to consume the desired substance, despite being aware of the negative consequences, is a defining criterion for addiction (American Psychiatric Association, 2013). Many 2BC models also involve intermittent periods of access to ethanol. This type of consumption/withdrawal cycle can produce escalating binge-like patterns of consumption, intoxication and withdrawal symptoms during periods of abstinence, which are also indicators of dependence (American Psychiatric Association, 2013; Carnicella et al., 2014).

Currently, the IA2BC and DID models are the only two voluntary consumption models available which can produce moderate to high levels of ethanol consumption in rodents without using sucrose-fading to induce ethanol consumption and are generally considered models of binge-like consumption. Wise (1973) first developed the IA2BC model. Simms et al. (2008) extended this work, showing the IA2BC model could be used to produce rats which consumed 20% ethanol at high levels (~5–6 g/kg/24 h), with approximately 30% of the rats achieving BECs around 90–100 mg/dL—meeting one of the National Institute on Alcohol Abuse and Alcoholism (NIAAA) criteria for binge drinking in humans (National Institute on Alcohol Abuse and Alcoholism (US) (2004)). Additionally, comparison of the IA2BC model with the traditional (non-binge-like) 10% continuous access 2BC model demonstrated a 40% increase in total ethanol consumption and improved sensitivity for detecting changes in ethanol consumption following administration of two different Federal Drug Administration (FDA) approved AUD medications, highlighting the importance of providing intermittent over continuous access to ethanol (Simms et al., 2008). The IA2BC model involves providing rats with access to one bottle of water and one of ethanol for 24 h on alternative days (Monday, Wednesday and Friday). Around the same time, work by Rhodes et al. (2005) demonstrated that the DID model could produce consumption of 20% ethanol at high levels (~2–3 g/kg/2 h and ~7 g/kg/4 h) and achieved blood ethanol concentrations (BECs) above 100 mg/dL in mice (Rhodes et al., 2005). The DID model provides mice with access to one bottle of water and one of ethanol for 2–4 h, 3 h into the dark phase of the light cycle, Monday to Friday.

Since the publication of both these models, the IA2BC model has predominantly been used to study ethanol consumption in rats and the DID for studies in mice, with both models demonstrating translation of discovered novel compounds into human clinical trials (for examples see Lhuintre et al., 1990; Mitchell et al., 2012). While the IA2BC is simple to use and accessible to most researchers, it has fewer consumption/withdrawal cycles per week (3 cycles/week) compared to the DID model (5 cycles/week), thereby offering fewer exposures to and withdrawals from ethanol per week in the same time-frame as the DID model. This becomes particularly important when conducting dose curve studies with novel compounds. Typically, the first day of the week is used to establish stable baseline drinking levels, the second day for the administration of one dose of the compound being examined and the third day to re-establish baseline consumption and eliminate any potential rebound effects. This means that the completion of dose curve studies using the IA2BC model can take over a month to complete as there are only three drinking sessions per week and only one dose can be tested per week. The DID model, on the other hand has five drinking sessions per week, producing more exposures in a shorter time period and potentially allowing two different doses to be tested per week. Using the IA2BC model for a dose curve study can result in significant differences in both the age of the rat and number of ethanol or sucrose exposures at which each dose is administered. While a latin square design is used to compensate for these differences, there is still the potential for increased noise and false negatives. Additionally, the dramatic rise in global rates of obesity and the subsequent burden of care faced by society has led to an increasing need for the development of rodent models for studying harmful sucrose consumption and its effects on the brain. Given the similarities between the effects of sucrose and other addictive substances on the reward pathway (Lemon et al., 2004; Spangler et al., 2004; Lenoir et al., 2007), ethanol consumption models are increasingly being adapted to study sucrose consumption. We recently utilized the IA2BC model in Wistar rats to show long-term binge-like sucrose consumption leads to morphological changes in the nucleus accumbens (Klenowski et al., 2016). We also demonstrated that varenicline, a partial agonist at α4β2 subunit containing nicotinic acetylcholine receptors, previously shown to reduce nicotine and ethanol consumption (Coe et al., 2005; Steensland et al., 2007), also reduces sucrose consumption (Shariff et al., 2016).

While ethanol and/or sucrose consumption studies have been conducted in mice using both the IA2BC and DID models and rats using the IA2BC model, it is currently unknown whether the DID model can be directly transferred to rats or if there are any benefits of using one model over the other for studying ethanol or sucrose binge-like consumption in rats. Specifically, it is unknown whether one model produces greater binge-like consumption than the other or if one model is more suitable for studying sucrose verses ethanol consumption behaviors. Here, we compare the DID and IA2BC models in Wistar rats consuming ethanol or sucrose. We explore whether the ethanol DID rat model meets the NIAAA binge drinking criteria. Additionally, we examine whether the reduced access time of the DID model impacts the efficacy of varenicline, a compound previously shown to reduce both ethanol and sucrose consumption using the rat IA2BC model (Steensland et al., 2007; Shariff et al., 2016).

## Materials and Methods

### Drugs

Five percent (w/v) sucrose (Sigma, ST. Louis, MO, USA) and 20% (v/v) ethanol solutions (Chem-supply, SA, Australia) were prepared in filtered water. Varenicline (6,7,8,9-tetrahydro-6,10-methano-6*H* pyrazino[2,3-*h*][3]benzazepine tartrate) was purchased from Tocris (Bristol, UK).

### Animals and Housing

Five week old male Wistar rats (193.7 ± 15.8 g; Animal Resource Centre, Perth, WA, Australia), were individually housed in ventilated Plexiglas cages (Tecniplast, Italy). The rats were acclimatized to the individual housing conditions, handling and reverse-light cycle for 5 days before the start of the experiments. All rats were housed in a climate-controlled 12-h reversed light cycle (lights off 9 am: lights on 9 pm) room with unlimited access to standard rat chow and water. The experimental procedures followed the ARRIVE guidelines (https://www.nc3rs.org.uk/arrive-guidelines) and were approved by the Queensland University of Technology Animal Ethics Committee and the University of Queensland Animal Ethics Committee, in accordance with the National Institutes of Health (NIH) guidelines for the care and use of laboratory animals.

### Intermittent-Access Two-Bottle Choice Model

**Figure 1:**
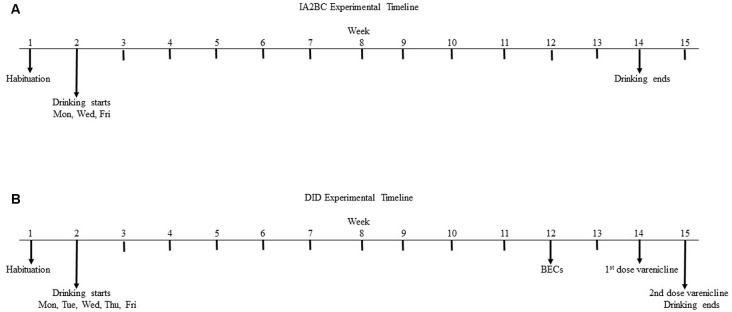
**Experimental timeline for the intermittent access 2-bottle choice (IA2BC) and drinking in the dark (DID) models.** Five week old male Wistar rats are given 1 week to habituate to the experimental housing conditions before commencing consumption of 20% ethanol or 5% sucrose using the IA2BC **(A)** or DID **(B)** models. Rats consuming ethanol using the DID model also had blood collected for blood ethanol concentration (BEC) measurement after 10 weeks of drinking sessions. After 12 weeks of ethanol or sucrose consumption, DID rats received 2 once weekly injections of vehicle or 2 mg/kg varenicline in pseudorandom order, such that each rat served as their own control.

The IA2BC drinking procedure was performed as previously described (Steensland et al., 2007; Shariff et al., 2016). All fluids were presented in 300-ml graduated plastic bottles with stainless-steel drinking spouts inserted through two grommets in the front of the cage immediately following the commencement of the dark phase of the light cycle (9 am). As previously reported, two bottles were presented simultaneously: one bottle containing water; the second bottle containing 5% (w/v) sucrose (Nielsen et al., 2008; Simms et al., 2011, 2012; Srinivasan et al., 2012; Klenowski et al., 2016; Patkar et al., 2016; Shariff et al., 2016) or 20% (v/v) ethanol (Steensland et al., 2007; Nielsen et al., 2008; Simms et al., 2008, 2010, 2012, 2014; Bito-Onon et al., 2011; Mill et al., 2013; Augier et al., 2014; Carnicella et al., 2014; Feduccia et al., 2014). The placement of the sucrose/ethanol bottle was alternated with each exposure to control for side preferences. Bottles were weighed 30 min, 2 h and 24 h after the fluids were presented, with measurements taken to the nearest 0.1 g. The weight of each rat was also measured to calculate the grams of sucrose or ethanol intake per kilogram of body weight. On the Monday after the end of the housing acclimatization period, rats (193.7 ± 15.8 g, *n* = 8 per group) were given access to one bottle of sucrose or ethanol and one bottle of water. After 24 h, the sucrose/ethanol bottle was replaced with a second water bottle that was available for the next 24 h. This pattern was repeated on Wednesdays and Fridays; all other days the rats had unlimited access to 2 bottles of water for 60 drinking sessions (see Figure 1A). For the sucrose experiments, due to technician error the measurements were not recorded at the 30 min and 2 h time points for the first 2 weeks of exposure (sessions 1–6, inclusive) and all measurements ceased after session 35 (*n* = 12). While measurements were not recorded the rats continued to have access to sucrose during this time. For the ethanol experiments, measurements were collected at all time points from session 1 through 60 (*n* = 8).

### Drinking in the Dark Two-Bottle Choice Model

The DID 2BC model was originally adapted from Rhodes et al. (2005) and modified to a 4 h daily access procedure for studying long-term ethanol consumption in mice by Santos et al. (2013). We used the methods described by Santos et al. (2013) in rats. The housing conditions, bottles and fluid concentrations were kept identical to the IA2BC procedure described above. However, the bottles were presented daily (Monday to Friday) for 4 h, 3 h following the commencement of the dark phase of the light cycle (12 pm) and bottle measurements were taken at 30 min, 2 h and 4 h after the fluids were presented. Drug administration began after the rats (*n* = 8 per group) had been consuming ethanol or sucrose for 10–12 weeks as the effects of ethanol and sucrose on the brain and the efficacy of varenicline are more robust following long term exposure (Klenowski et al., 2016; Patkar et al., 2016; Shariff et al., 2016). As previously described (Steensland et al., 2007; Patkar et al., 2016; Shariff et al., 2016), varenicline (2 mg/kg) or vehicle (0.9% saline) were administered by subcutaneous (s.c.) injection, 30 min before the drinking session, once per week over 2 weeks at a volume of 1 ml/kg using a Latin square design, such that each rat received both doses and served as their own control. For both the ethanol (*n* = 8) and sucrose (*n* = 8) experiments, all measurements were collected at all time points from session 1 through 65 but only sessions 1–50 are used to assess baseline consumption due to blood collection in week 10 and drug testing in weeks 12 and 13 (see Figure 1B).

### Blood Ethanol Measurements

The rats were anesthetized with isoflurane and tail blood collected 30 min after bottle presentation in the 10th week of ethanol consumption. Approximately 100 μl of whole blood was collected into tubes containing 10 μL of 2.5 M EDTA. The blood was centrifuged at 4°C for 20 min at 1500× g and the serum aliquoted and stored at −80°C until assayed. BECs were determined using the nicotinamide adenine dinucleotide (NAD)—alcohol dehydrogenase (ADH) spectrophotometric assay as previously described (Zapata et al., 2006; Santos et al., 2013). All reagents used in this assay were purchased from Sigma-Aldrich (St. Louis, MO, USA). All samples and standards were run in triplicate and BECs were calculated using a standard calibration curve.

### Statistics

Statistical analysis was performed using GraphPad Prism software (version 6, USA) and all results are expressed as mean ± standard error of the mean (SEM). Unpaired *T*-test with Holm-Sidak method (alpha value adjusted to 0.017 using Sidak correction) was used to compare consumption at each time point and to compare vehicle and varenicline treatments at each time point. Unpaired, two-tailed Student’s *T*-test was used to compare hourly and weekly consumption. Linear Regression and Pearson’s *r* coefficient were performed for the BEC and ethanol consumption of each DID rat. Multiple *t*-tests with Holm-Sidak correction (alpha = 0.010) were used to compare consumption at each time point, per week and per hour for each model and reinforcer.

## Results

### Comparing Ethanol Consumption per Drinking Session Using the IA2BC and DID Models

**Figure 2:**
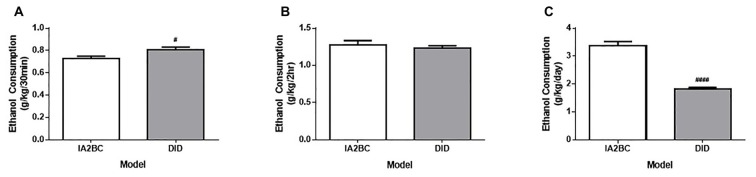
**Comparison of ethanol consumption per session using the IA2BC and DID models. (A)** Rats using the DID model (gray) consumed more 20% ethanol than IA2BC rats (white) following 30 min of ethanol access. **(B)** However, following 2 h of ethanol access, both the DID and IA2BC rats consumed similar amounts of ethanol. **(C)** The total amount of ethanol consumed per session was greater for rats using the IA2BC model compared to the DID model. *n* = 8. Unpaired *T*-tests with Holm-Sidak correction (alpha = 0.017). ^#^*p* < 0.017, ^####^*p* < 0.0001 compared to IA2BC rats.

To determine whether the DID model could be used to induce high ethanol consumption in rats, we compared the average ethanol consumption per drinking session for the first 50 sessions in rats using the DID model with rats using the established IA2BC model (for review of IA2BC model see Carnicella et al., 2014). In this study, the average amount of ethanol consumed in the first 30 min was greater for DID rats (0.73 ± 0.077 g/kg/30 min) compared to IA2BC rats (0.81 ± 0.074 g/kg/30 min; Figure 2A, unpaired *T*-test with Holm-Sidak correction, *p* = 0.0130, *n* = 8). The average amount of ethanol consumed after 2 h ethanol access was similar for IA2BC and DID rats (1.28 ± 0.098 g/kg/2 h and 1.23 ± 0.093 g/kg/2 h, respectively; Figure 2B, *T*-test with Holm-Sidak correction, *p* = 0.5087, *n* = 8). However, the average amount of ethanol consume during the entire drinking session was greater for IA2BC rats (3.34 ± 0.362 g/kg/24 h) compared to DID rats (1.83 ± 0.140 g/kg/4 h; Figure 2C, unpaired *T*-test with Holm-Sidak correction, *p* < 0.0001, *n* = 8).

### DID and IA2BC Rats Consume Similar Amounts of Ethanol per Week

**Figure 3:**
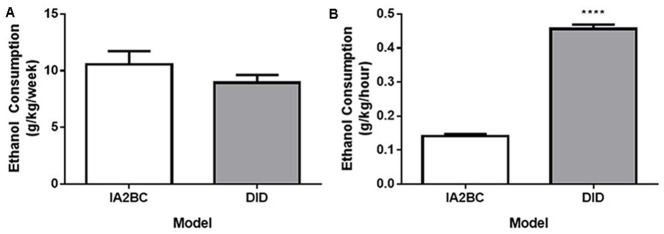
**Comparison of weekly and hourly ethanol consumption using the IA2BC and DID models. (A)** Rats consuming 20% ethanol using the DID (gray) and IA2BC (white) model consumed similar amounts of ethanol per week. **(B)** However, the average amount of ethanol consumed per hour was greater for rats using the DID model compared to the IA2BC model *n* = 8 per group. Two tailed unpaired Student’s *T*-test. ****p < 0.0001 compared to IA2BC rats.

Although the rats using the IA2BC model consumed more ethanol per session compared to the DID model, each model possessed differences in the amount of time available per session to access ethanol and the number of drinking sessions per week. First, different lengths of time were needed to reach the same number of drinking sessions for each model. For example, the IA2BC rats needed to drink for 10 weeks to complete 30 sessions whereas the DID rats only needed to drink for 6 weeks to complete the same number of drinking sessions. Second, the amount of time the rats were able to access the ethanol per week was different between the models: the IA2BC rats had 3 × 24 h sessions (72 h) per week compared to 5 × 4 h sessions (20 h) per week for the DID rats. To determine how the weekly differences in ethanol access (session time and number) affected the total amount of ethanol the rats received, we compared the average amount of ethanol consumption per week (Figure 3A). We also compared the average amount of ethanol consumed per hour (Figure 3B) for each model. Using unpaired two-tailed Student’s *T*-test we found no difference in the amount of ethanol consumed per week (IA2BC: 10.56 ± 1.163 g/kg/week and DID: 8.94 ± 0.687 g/kg/week, *p* = 0.2626, *n* = 8). However, the amount of ethanol consumed per hour was greater for the DID rats (0.46 ± 0.013 g/kg/h) compared to IA2BC rats (0.14 ± 0.007 g/kg/h; unpaired two-tailed Student’s *T*-test, *p* < 0.0001, *n* = 8).

### Assessing the DID Model Against NIAAA Binge Drinking Criteria

**Figure 4:**
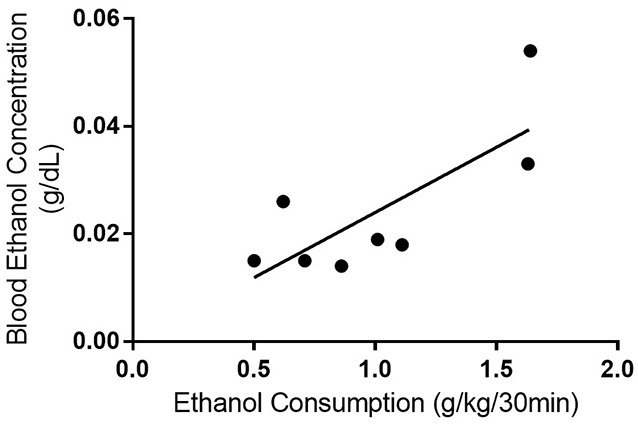
**Blood ethanol consumption correlates with ethanol consumption with the DID model.** Linear regression and Pearson’s correlation coefficient were used to show that BEC increased with ethanol consumption in rats using the DID model (*r^2^* = 0.591, *p* = 0.026). *n* = 8 per group.

In 2004, the NIAAA set out criteria for defining binge drinking in humans. A binge drinking session was defined as a pattern of drinking alcohol that causes a blood alcohol concentration of at least 80 mg/dL, which equates to approximately five or more drinks consumed by an adult male in 2 h (National Institute on Alcohol Abuse and Alcoholism (US) (2004)). We estimated that an average adult male would need to consume around 0.87 g/kg in 2 h to meet the criteria of binge drinking based on one standard drink containing 14 g of pure alcohol (CDC, 2016) and 80.7 kg for the average body weight of an adult in North America (Walpole et al., 2012). However, we could not find similar criteria for binge-like consumption for rodents in the literature and it appears most rodent models of consumption have been designed based on human criteria. As such, we were limited to comparing the rodent consumption from each model with the human binge criteria. Both the IA2BC and DID rats consumed more than 0.87 g/kg during the first 2 h of their ethanol consumption sessions (IA2BC: 1.28 ± 0.098 g/kg/2 h and DID: 1.23 ± 0.093 g/kg/2 h). Next, we collected blood from the DID rats to determine whether they reached BECs of 80 mg/dL or greater (BECs for IA2BC rats have been reported previously, see Simms et al., 2008). We measured BECs 30 min after the commencement of a standard drinking session during the 10th week of ethanol consumption (as previously published Steensland et al., 2007; Simms et al., 2008). Thirty minute BECs were chosen over 2 h BEC measurements as rodent metabolic rates are different to humans and previous studies indicate peak BECs using the IA2BC model are achieved by this time point in rodents (Simms et al., 2008; Cippitelli et al., 2012; George et al., 2012). We compared 30 min BECs with the amount of ethanol consumed during the 30 min prior to blood collection (Figure 4). The average BEC was 24.45 ± 4.826 mg/dL and BECs ranged from 14.24 to 54.00 mg/dL. Ethanol consumption in the 30 min prior to blood collection was 1.01 ± 0.153 g/kg/30 min, ranging from 0.50 to 1.64 g/kg/30 min. There was a significant correlation of BECs with ethanol consumption (*R*^2^ = 0.598, *p* < 0.05). However, none of the DID rats BECs met the NIAAA binge drinking criteria of 80 mg/dL after 30 min of ethanol access.

### Varenicline Reduces Ethanol Consumption in DID Rats

**Figure 5:**
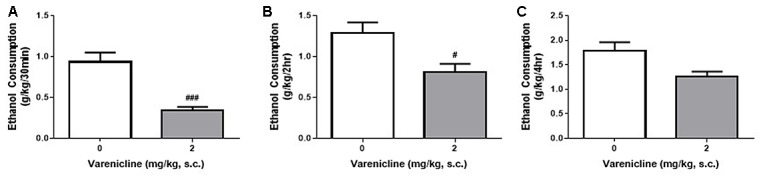
**Varenicline reduces ethanol consumption in DID rats.** Administration of 2 mg/kg varenicline (gray) produced a significant reduction in ethanol consumption 30 min **(A)**, 2 h **(B)** but not 4 h **(C)** after the commencement of a standard DID drinking session compared to vehicle (white). *n* = 8 per group. Unpaired *T*-tests with Holm-Sidak correction (alpha = 0.017). ^#^*p* < 0.017, ^###^*p* < 0.001 compared to vehicle.

Despite the differences in ethanol consumption per session, the DID and IA2BC models resulted in the rats receiving similar amounts of ethanol each week and a higher initial and hourly consumption rate in DID rats. Therefore, to examine whether the differences in consumption produced by each model and the differences in ethanol access times affected model suitability for pre-clinical screening of novel compounds as potential treatments for controlling ethanol consumption, we next assessed whether the DID model could detect reductions in ethanol consumption following treatment with varenicline. Using the IA2BC model in rats, we have previously shown that varenicline (2 mg/kg) reduces ethanol consumption following 30 min and 6 h of ethanol access (Steensland et al., 2007). In this study using DID rats, varenicline also significantly reduced ethanol consumption compared to vehicle at 30 min (Figure 5A) and 2 h (Figure 5B) but not 4 h (Figure 5C) following bottle presentation (Unpaired *T*-test with Holm-Sidak correction, 30 min: *p* = 0.0003, 2 h: *p* < 0.0101, 4 h: *p* < 0.0232, *n* = 8).

### Daily Sucrose Consumption Using the IA2BC and DID Models

**Figure 6:**
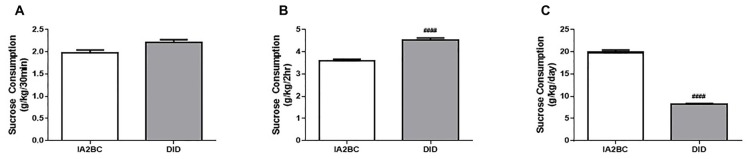
**Comparison of sucrose consumption per session using the IA2BC and DID models.** Using the DID model (gray), rats consumed similar amounts of 5% sucrose than rats using the IA2BC model (white) following 30 min **(A)** and more sucrose after 2 h **(B)** of sucrose access. **(C)** However, the total amount of sucrose consumed per session was greater for rats using the IA2BC model compared to the DID model *n* = 8–12 per group. Unpaired *T*-tests with Holm-Sidak correction (alpha = 0.017). ^####^*p* < 0.0001 compared to IA2BC rats.

To determine whether the DID model could also be used to study sucrose binge-like consumption in rats, we compared daily sucrose consumption using the IA2BC and the DID model. As demonstrated by Srisontiyakul et al. (2016), a significantly lower concentration of sucrose is required to produce similar reward seeking behaviors to ethanol, therefore 5% rather than 20% sucrose was presented to the rats during drinking sessions. We have recently shown long-term (10 weeks) consumption of 5% sucrose using the IA2BC model leads to morphological changes in the brain (Klenowski et al., 2016), indicating suitability of this procedure for modeling the effects of long-term binge-like sucrose consumption on the brain. Analysis using the unpaired *T*-test with Holm-Sidak correction revealed that IA2BC rats consumed similar amounts of sucrose than DID rats after 30 min but less sucrose compared to DID rats after 2 h of sucrose access (30 min: Figure 6A, *p* = 0.0171 and 2 h: Figure 6B, *p* < 0.0001). However, the IA2BC rats consumed more sucrose per session than DID rats (Figure 6C, *p* < 0.0001). The average amount of sucrose consumed using the IA2BC model was 1.98 ± 0.085 g/kg/30 min, 3.58 ± 0.198 g/kg/2 h and 19.85 ± 0.881 g/kg/24 h and for the DID model it was 2.21 ± 0.138 g/kg/30 min, 4.52 ± 0.220 g/kg/2 h and 8.19 ± 0.410 g/kg/4 h.

### The IA2BC Model Induces Greater Total Sucrose Intake per Week

**Figure 7:**
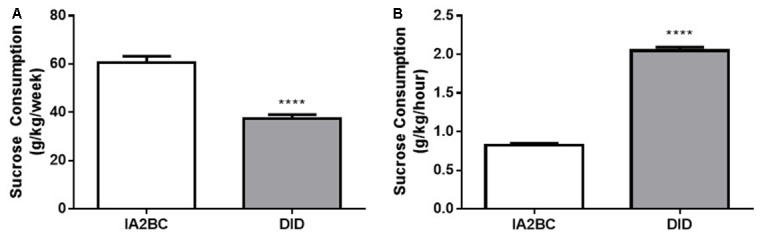
**Comparison of weekly and hourly sucrose consumption using the IA2BC and DID models. (A)** Using the IA2BC model (white), rats consumed more 5% sucrose per week compared to rats using the DID model (gray). **(B)** However, the average hourly sucrose intake was greater for DID rats compared to IA2BC rats. *n* = 8–12 per group. Unpaired two-tailed Student’s *T*-test. ****p < 0.0001.

Next, to further examine the sucrose consumption produced by each model, we compared the average amount of sucrose consumed per week (Figure 7A) and the amount of sucrose consumed per hour (Figure 7B). Using the unpaired, two-tailed Student’s *T*-test we found rats using the IA2BC model consumed more sucrose per week (*p* < 0.0001) but less sucrose per hour (*p* < 0.0001) compared to DID rats. The IA2BC rats consumed 60.50 ± 2.731 g/kg of sucrose per week and 0.83 ± 0.022 g/kg per hour whereas the DID rats consumed 37.30 ± 1.727 g/kg of sucrose per week and 2.05 ± 0.045 g/kg per hour.

### Varenicline also Reduces Sucrose Consumption Using the DID Model

**Figure 8:**
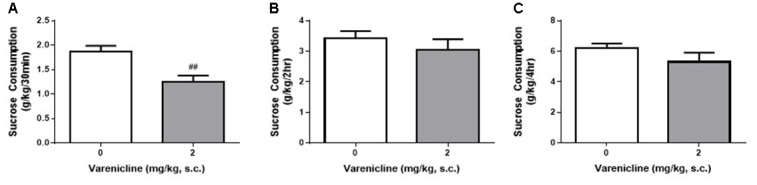
**Varenicline reduces sucrose consumption in DID rats following 30 min of sucrose access. (A)** Administration of 2 mg/kg varenicline (gray) produced a significant reduction in sucrose consumption compared to saline (white) following 30 min of sucrose access using the DID model. Varenicline had no effect on sucrose consumption at the 2 h **(B)** and 4 h **(C)** time points *n* = 8 per group. Unpaired *T*-tests with Holm-Sidak correction (alpha = 0.017). ^##^*p* < 0.01 compared to vehicle.

Given the differences daily, weekly and hourly consumption in the DID and IA2BC rats, we wanted to know whether the reduced access times of the DID model impacted the ability to detect varenicline-induced reductions in sucrose consumption. We have previously shown, using the IA2BC model, that varenicline (2 mg/kg, s.c.) reduces sucrose consumption following 30 min but not 2 h of sucrose access (Shariff et al., 2016). Similar to our previous IA2BC study, varenicline significantly reduced sucrose consumption compared to vehicle after 30 min (Figure 8A) but not after 2 h (Figure 8B) or 4 h (Figure 8C) of sucrose access in DID rats (Unpaired *T*-test with Holm-Sidak correction, 30 min: *p* = 0.0050, 2 h: *p* = 0.4095 and 4 h: *p* = 0.1889, *n* = 6).

### Comparison of Consumption between Models and Reinforcers

To further explore the effect each model had on consumption of ethanol and sucrose, we compared ethanol to sucrose consumption produced by each model using multiple *t*-tests with Holm-Sidak correction (alpha = 0.010). We compared consumption after 30 min and 2 h, as well as per session, weekly and hourly (Table 1). For both models, sucrose consumption was significantly greater than ethanol consumption (*p* < 0.0001) for all consumption parameters examined. Re-examination of each of these parameters for each reinforcer (see Table 1) with this statistical method produced similar results to those presented above. Ethanol consumption was greater per session for the IA2BC model (*p* < 0.001) but ethanol consumption per hour was greater with the DID model (*p* < 0.0001). Both models produced similar sucrose consumption after 30 min and 2 h. While sucrose consumption was greater per session (*p* < 0.001) and per week (*p* < 0.0001) using the IA2BC model, like ethanol consumptions, hourly sucrose consumption was greater using the DID model (*p* < 0.0001).

**Table 1:** **Comparison of ethanol and sucrose consumption for each model and reinforcer**.

	IA2BC		DID	
**Consumption**	**Ethanol**	**Sucrose**	**Ethanol**	**Sucrose**
g/kg/30 min	0.81 ± 0.074	1.98 ± 0.085****	0.73 ± 0.077	2.21 ± 0.138^####^
g/kg/2 h	1.28 ± 0.098	3.58 ± 0.198****	1.23 ± 0.093	4.52 ± 0.220^####^
g/kg/session	3.34 ± 0.362	19.85 ± 0.881****	1.83 ± 0.140^$$$^	8.19 ± 0.410^####,&&&^
g/kg/week	10.56 ± 1.163	60.50 ± 2.731****	8.94 ± 0.687	37.30 ± 1.727^####,&&&&^
g/kg/hour	0.14 ± 0.007	0.83 ± 0.022****	0.46 ± 0.013^$$$$^	2.05 ± 0.045^####,&&&&^

For each model and reinforcer multiple t-tests with Holm-Sidak correction (alpha = 0.010) were performed; n = 8–12 per group; ****p < 0.0001 IA2BC ethanol compared to IA2BC sucrose; ^####^p < 0.0001 DID ethanol compared to IA2BC Sucrose; ^$$$^p < 0.001, ^$$$$^p < 0.0001 IA2BC ethanol compared to DID ethanol; ^&&&^p < 0.001, ^&&&&^p < 0.0001 IA2BC sucrose compared to DID sucrose.

## Discussion

In this study, we demonstrate for the first time that rats will consume moderate to high amounts of 20% ethanol and 5% sucrose using the DID model, a model traditionally used with mice. We compared their consumption to rats using the IA2BC model and found they produced different consumption patterns for ethanol and sucrose (see Table 1). While the IA2BC rats consumed more ethanol per session than DID rats, the DID rats displayed similar weekly consumption and higher initial and hourly consumption of ethanol. Together, this suggested that despite the IA2BC rats receiving 52 h more access to ethanol per week, the DID model produced greater binge-like consumption, particularly during the first 30 min of the drinking session. Similarly, The IA2BC rats consumed more sucrose per session and the DID rats displayed greater initial and hourly sucrose consumption. However, unlike ethanol, the IA2BC rats displayed greater weekly consumption of sucrose. Looking at ethanol and sucrose consumption across time during a standard drinking session for each model, DID rats consumed more ethanol than IA2BC rats at 30 min but at 2 h their consumption was similar. For sucrose the DID rats consumed similar amounts of sucrose at 30 min and more sucrose at 2 h. This may imply that the rate of sucrose consumption remains fairly consistent with time for both models, and while the DID model appears to induce greater binge-like sucrose consumption, the increased access time may be beneficial for studying the effects of long-term excessive sucrose consumption on the brain as it ensures exposure to greater amounts of sucrose. However, the data presented in this study indicate that the rate of ethanol consumption declines as access time increases and the rate of decline is more rapid for the DID compared to the IA2BC model. As such, the longer period of access to ethanol provided by the IA2BC model may not provide any additional benefits for studying binge-like consumption or the effects of long-term excessive ethanol consumption.

The differences in ethanol and sucrose consumption behaviors (discussed above) are, most likely, a reflection of the sedating effects of ethanol. Ethanol has both stimulatory and sedating effects depending on the dose administered. At low doses, ethanol increases locomotor activity, whereas higher doses reduce locomotor activity and can lead to loss of consciousness (June et al., 2003; Zapata et al., 2006; Kawakami et al., 2007; Arias et al., 2012; Kippin, 2014; Botia et al., 2015; Karlsson and Roman, 2016). As such, the more rapidly ethanol is consumed and the greater the intake, the more sedated the rat is likely to become, and the length of time the rat would be capable of engaging in the further consumption of ethanol would be reduced. Certainly, the elevated initial and hourly ethanol consumption of the DID model combined with its more rapid decline in ethanol consumption compared to the IA2BC model indicate not only a greater propensity to induce binge-like consumption but also a greater level of impairment. However, our 30 min BEC data do not support greater levels of impairment with the DID model compared to the IA2BC model and it may be that 30 min BEC measures is not suitable for use in rodents given their high metabolic rates (discussed further below). Other measures of motor impairment (for example, locomotor activity, rotarod, footprint analysis) are required to determine whether one model produces greater intoxication over the other. Sucrose exposure, on the other hand, does not appear to alter locomotor activity (Avena and Hoebel, 2003) and therefore, continued consumption of sucrose would not be expected to alter the ability to continue consumption. Measurements of locomotor activity during the drinking sessions are needed to confirm this, particularly as there is little data available regarding the effects of sucrose on locomotor activity. Another possibility is that the differences in consumption patterns observed result from differences in the types of neuronal pathways that ethanol and sucrose are capable of manipulating (for example, the reward pathway for ethanol [for review, see Söderpalm and Ericson, 2013) vs. the appetitive and reward pathways (Spangler et al., 2004) for sucrose], as well as differences in the amount of neurotransmitters released (Doyon et al., 2004; Funk and Dohrman, 2007) and the subtypes of receptors involved (Hodge et al., 1994). Unfortunately, many studies conducted which investigate these factors have compared sucrose consumption with sucrose-sweetened ethanol solutions (Hodge et al., 1994; Slawecki et al., 1997; Doherty and Gonzales, 2015) making it difficult to isolate the effects of ethanol alone. Future research is likely to address this now that voluntary 2BC models are available that do not require sucrose-fading to induce higher levels of ethanol consumption.

As a large proportion of the total ethanol consumed occurred during the initial period of the drinking session and the rate of ethanol consumed per hour for the DID model was higher than the IA2BC model (see Table 1), we examined the rat DID model to see if it could be defined as a model of human binge alcohol consumption. The first of the NIAAA defined criterion required the consumption of five or more standard drinks within 2 h (National Institute on Alcohol Abuse and Alcoholism (US) (2004)). We estimated that this was the equivalent of 0.87 g/kg in 2 h for the average adult male. The ethanol consumption for the IA2BC and DID models easily exceeded this criterion, suggesting both procedures effectively model binge drinking. However, we were unable to achieve the second NIAAA criterion for binge drinking: BECs at or above 80 mg/dL (National Institute on Alcohol Abuse and Alcoholism (US) (2004)) after 30 min of access to ethanol. Previously, we reported that Wistar rats and alcohol preferring (P) rats consuming 20% ethanol with the IA2BC model achieved BECs 4–93 mg/dL and 11–63 mg/dL (respectively) after 30 min of ethanol access (Simms et al., 2008). While approximately 30% of the Long Evans rats in the study achieved BECs above 90 mg/dL, only one of the Wistar and none of the alcohol preferring (P) rats achieved a BEC above 80 mg/dL using the IA2BC model (Simms et al., 2008). The range of the DID rats BECs (14.24–54.00 mg/dL) in this study was consistent with the previously reported BECs obtained using the IA2BC model. Based on this it seemed possible, regardless of the model used, 30 min may not be sufficient time for the rats to drink enough alcohol to achieve a BEC of 80 mg/dL. However, data from other IA2BC studies using Wistar rats suggest that peak BECs occur by 30 min and increasing the length of ethanol access prior to BEC measurement does not increase the BEC obtained. Cippitelli et al. (2012) reported that Wistar rats consuming ethanol for 1 h had BECs ranging from 7 mg/dL to 61 mg/dL after consuming around 5.2 g/kg of ethanol. Extending the ethanol access time to 2 h produced BECs of approximately 58 mg/dL following consumption of about 3.6 g/kg of ethanol (George et al., 2012). A more likely possibility is that the higher metabolic rate and smaller body size of rats (compared to humans) makes it difficult for the rats to consume a sufficient amount of ethanol to reach the 80 mg/dL defined for humans in the NIAAA binge drinking criteria. Some studies with mice have utilized pyrazole, an alcohol dehydrogenase (ADH) inhibitor, to prevent ethanol metabolism, and allow more consistent BEC measurement (Terdal and Crabbe, 1994; Griffin et al., 2009). Further studies are required to determine the time after ethanol access commences that peak BECs occur and to explore whether the use of pyrazole is required for rat BEC studies. Whether a BEC of 80 mg/dL or more is an appropriate measurement for classifying binge drinking in rodents also remains to be explored.

Finally, we assessed whether the rat DID model could be used to measure changes in ethanol and sucrose consumption following treatment with varenicline, a compound previously shown to reduce both ethanol and sucrose consumption using the IA2BC model in Wistar rats (Steensland et al., 2007; Shariff et al., 2016). Given the differences in the number of drinking sessions per week and ethanol and sucrose access times between the IA2BC and DID models, we chose to use varenicline at a dose of 2 mg/kg as we have previously shown this dose significantly reduced ethanol (Steensland et al., 2007) and sucrose (Shariff et al., 2016) consumption following two different lengths of exposure (4 and 12 weeks), whereas the lower dose of 1 mg/kg was only effective following 12 weeks of exposure to ethanol. Similar to previously demonstrated with the IA2BC model, varenicline reduced consumption at the 30 min and 2 h time points for ethanol and only at the 30 min time point for sucrose. However, we failed to demonstrate a significant effect at the 4 h time point for Varenicline on ethanol consumption, which we had shown with the IA2BC model (Steensland et al., 2007). Most likely this is due to differences in the statistical analysis as the previous study did not correct for multiple *t*-tests. In the present study, the *p* value was less than 0.05 at 4 h and without the alpha value correction this could be considered significant. While there is the possibility that use of the alpha adjustment increases the amount of type II error, it is generally considered the more suitable statistical test as it reduces the amount of type I error when using multiple *t*-tests. Considering this, the level of significance achieved was also similar to that obtained with the IA2BC model for both ethanol and sucrose, indicating that the DID model may have similar sensitivity to the IA2BC model for screening novel compounds. Further testing with lower doses, additional time points and other compounds known to reduce ethanol and sucrose consumption is necessary to determine whether this is indeed the case or if one model of binge-like consumption offers greater sensitivity than the other, particularly for sucrose consumption. Although, the fact that we were able to detect a reduction in both sucrose and ethanol consumption with varenicline indicates that despite the reduced access time, the increased number of ethanol exposures and greater initial and hourly binge-like consumption produced by the DID model may be sufficient to produce signaling changes within the brain, similar to that reported with the IA2BC model (see Nielsen et al., 2012a; Klenowski et al., 2016). Further studies are also required to determine which aspects of the model (i.e., the increased number of consumption/withdrawal cycles of the DID model verses increased total amount consumed per session of the IA2BC model) are more important for modeling and producing binge-like consumption, if the DID model produces brain changes similar to those previously demonstrated with the IA2BC model for sucrose and ethanol consumption and whether these changes occur following fewer weeks of ethanol or sucrose exposure compared to the IA2BC model.

## Conclusion

The DID model is adaptable for use as a rat model of both ethanol and sucrose binge-like consumption. It produces higher initial and hourly consumption of ethanol and sucrose compared to the IA2BC model, suggesting it induces greater binge-like consumption. Despite the lower total consumption per session produced by the DID model, it detected varenicline-induced reductions in both ethanol and sucrose consumption, similar to that reported for the IA2BC model. Further studies are required to dissect the importance of the number of exposures, length of access and total amount consumed for modeling consumption behaviors. However our data suggest that the DID model may offer advantages over the IA2BC model, particularly for studying binge-like consumption of ethanol. Further examination of the differences produced by each model is required to provide greater guidance for researchers when assessing the suitability of each model for their study.

## Author Contributions

JYH made substantial contributions to the conception and design of the work, the acquisition, analysis and interpretation of data for the work, drafting the work and revising it critically for important intellectual content. MS and EWHM made substantial contributions to the acquisition and analysis and revising the work critically for important intellectual content. SB made substantial contributions to the design of the work, interpretation of data for the work and revising the work critically for important intellectual content. All authors gave final approval of the version to be published and agreement to be accountable for all aspects of the work in ensuring that questions related to the accuracy or integrity of any part of the work are appropriately investigated and resolved.

## Funding

This work was supported by funding from the Australian Research Council (Grant No. FT1110884), the National Health and Medical Research Council (Grant No. 1049427) and the National Institute of Health (Grant No. NS59910).

## Conflict of Interest Statement

The authors declare that the research was conducted in the absence of any commercial or financial relationships that could be construed as a potential conflict of interest.

## Abbreviations

ADE: alcohol deprivation effect
AUD: alcohol use disorder
BEC: blood ethanol concentration
CDC: Center for Disease Control and Prevention
DID: drinking in the dark
FDA: Federal Drug Administration
IA2BC: intermittent access 2-bottle choice
NIAAA: National Institute on Alcohol Abuse and Alcoholism
NIH: National Institutes of Health
s.c.: subcutaneous
WHO: World Health Organization.
